# Supplementary material for: Approach in inputs & outputs selection of Data Envelopment Analysis (DEA) efficiency measurement in hospitals: A systematic review
Source: PLoS One. 2024 Aug 14;19(8):e0293694. doi: 10.1371/journal.pone.0293694 (PMC11324144; doi:10.1371/journal.pone.0293694)
Supplement: S3 Appendix — (DOCX) [file pone.0293694.s003.docx]

Appendix C

**Table 6**

Summary of 89 reviewed publications

| **Author(s) (year)** | **Hospital types** | **Number of hospitals** | **Number of DMU(s)** | **Model parameters** | | | | | **Efficiency types** | **Inputs & Outputs selection approach** |
| --- | --- | --- | --- | --- | --- | --- | --- | --- | --- | --- |
|  |  |  |  | **Model types** | **Return to scale** | **Model orientation** | **Inputs** | **Outputs** |  |  |
| (Czypionka et al., 2014) | Public & Private Hospitals | 128 | 128 | BCC | VRS | Input | No. of physicians  No. of nurses  No. of medical staffs  No. of other staffs  Capital costs  Operating costs  Secondary costs | Outpatient visits  DRG credits | PTE | Literature review |
| (Fragkiadakis et al., 2014) | Public General Hospital | 87 | 348 | CCR, BCC | CRS, VRS | Input | No. of nurses  No. of medical staffs  No. of administrative staffs  Personnel costs  Supplies costs | No. of outpatient & emergency visits  Inpatient days  No. of surgeries  No. of lab examinations | TE, PTE, SE | Literature review |
| (Özgen Narcı et al., 2014) | Public General Hospital | 1089 | 1089 | BCC | VRS | Input | No. of specialists  No. of general practitioners  No. of nurses  No. of other staffs  No. of actual beds | No. of outpatient  No. of emergency visits  No. of discharges  No. of surgeries  No. of daycare patients | PTE | Literature review |
| (Cavalieri et al., 2014) | All Hospitals | 492 | 5904 | CCR, BCC | CRS, VRS | Input | No. of physicians  No. of nurses  No. of other staffs  No. of beds | Inpatient days  No. of discharges  CaseMix adjusted discharges | TE | Literature review |
| (Matranga & Sapienza, 2015) | Acute Care Hospital | 116 | 116 | Congestion, CCR, BCC | CRS, VRS | Output | No. of doctors  No. of nurses  No. of other staffs  No. of beds | No. of inpatient admission  CaseMix adjusted discharges | TE, PTE, SE | Literature review |
| (Gok & Altındağ, 2015) | Public & Private Hospitals | 477-741 | 4717 | BCC | VRS | Input | No. of specialists  No. of non-specialist  No. of beds | No. of outpatient  ALOS  BOR  BTR  No. of surgeries  No. of birth deliveries  No. of discharges | PTE | Literature review |
| (H. Li & Dong, 2015) | Public General Hospital | 14 | 14 | CCR | CRS | Output | No. of hospital staffs  No. of actual beds | No. of discharges  No. of diagnostic visits | TE | Literature review (local studies) |
| (Cheng et al., 2015) | District Hospital | 114 | 342 | CCR, BCC | CRS, VRS | Input | No. of physicians  No. of nurses  No. of actual beds | No. of outpatient & emergency visits  Inpatient days | TE, PTE, SE | Literature review & Data availability |
| (Tiwari & Shukla, 2015) | Not Specified | NA | NA | SBM, CCR, BCC | CRS, VRS | Output | FTE physicians  FTE nurses | No. of outpatients  No. of inpatients  Total revenues | TE, PTE, SE | Literature review |
| (Rezaee & Karimdadi, 2015) | All Hospitals | 288 | 31 | CCR | CRS | NA | No. of hospital staffs  No. of actual beds  No. of medical equipment | No. of outpatients  No. of inpatients  No. of special patients  BOR  Bed-days | TE | Literature review |
| (Almeida et al., 2015) | Public General Hospital | 37 | 37 | BCC | VRS | Output | No. of doctors  No. of nurses  No. of other staffs  No. of beds  Total costs | No. of outpatients  No. of emergency visits  CaseMix adjusted inpatients  No. of ambulatory surgeries  No. of non-ambulatory surgeries | PTE | Literature review |
| (Chowdhury & Zelenyuk, 2016) | All Hospitals | 113 | 226 | CCR | CRS | Output | FTE nurses  FTE administrative staffs  No. of actual beds  Medical & surgical costs  Equipment costs  Non-medical costs | CaseMix adjusted inpatient days  Ambulatory visits | TE | Literature review & Data availability |
| (van Ineveld et al., 2016) | Public General Hospital | 59-65 | 59-65 | CCR, BCC | CRS, VRS | Input | FTE physicians  FTE non-physicians  Operating costs | No. of outpatient 1^st^ visits  No. of admission  No. of daycare treatment | TE, PTE, SE | Literature review |
| (Villalobos-Cid et al., 2016) | Public Hospital | 193 | 193 | CCR, BCC | CRS, VRS | Output | Human resource expenses  Services & goods expenses | Inpatient days  No. of discharges  No. of outpatient appointment | TE | Literature review & Variance filter method |
| (Wang et al., 2016) | Maternal and Child Health Hospitals | 32 | 32 | BCC | VRS | Input | No. of doctors  No. of nurses  No. of actual beds  Total expenditure | No. of outpatient & emergency visits  No. of discharges  Total revenues | PTE | Literature review |
| (Str & Kalogeropoulou, 2016) | Public General Hospital | 90 | 90 | CCR, BCC | CRS, VRS | Input | No. of physicians  No. of nurses & administrative staffs  No. of beds  Total expenditure | No. of inpatients  No. of surgeries | TE, PTE, SE | Literature review |
| (ArulJothi, K et al., 2016) | Public Hospital | NA | 35 | CCR | CRS | NA | No. of specialists  No. of doctors  No. of health assistants  No. of health workers  No. of beds  No. of hospitals  Centre types | Average population served per hospitals  Average population served per hospital beds  Projected population | TE | Literature review |
| (R. C. Li et al., 2016) | Public Hospital | 14 | 14 | Additive | NR | Both | No. of hospital staffs  No. of beds  Total expenditure  No. of population | No. of total patients  No. of lab services  Death rate | TE | Literature review & DMU limitation |
| (Caballer-Tarazona et al., 2016) | Public & Private Hospitals | 24 | 24 | NA | NA | NA | No. of beds  No. of operating rooms  Staff costs  Other costs | CaseMix adjusted inpatients  No. of adjusted outpatient  No. of adjusted surgical patients  Management score | NA | Literature review |
| (Mujasi et al., 2016) | Public & Private (Non-profit) Hospitals | 17 | 17 | CCR, BCC | CRS, VRS | Output | No. of medical staffs  No. of beds | No. of outpatients  Inpatient days | TE, PTE, SE | LR* & Data availability |
| (Flokou et al., 2017b) | Public General Hospital | 71 | 71 | CCR, BCC | CRS, VRS | Input | FTE doctors  FTE other staffs  No. of beds | No. of outpatients  No. of inpatients  No. of surgeries | TE, PTE, SE | Literature review |
| (Valdmanis et al., 2017) | Acute Care Hospital | 43 | 215 | CCR | CRS | Input | FTE physicians & dentists  FTE nurses  FTE other staffs  No. of actual beds | No. of all outpatients  No. of inpatients | TE | Literature review |
| (Soares et al., 2017) | Public Hospital | 21 | 21 | SBM | NR | Non-oriented | No. of medical & non-medical staffs  No. of beds  ALOS  Annual revenues | No. of outpatients  No. of admission  No. of surgical operations  No. of clinical examination | TE | Literature review |
| (Jiang et al., 2017) | Public General Hospital | 1105 | 2210 | CCR, BCC | CRS, VRS | Input | No. of physicians  No. of nurses  No. of medical staffs  No. of actual beds | No. of outpatient & emergency visits  Inpatient days | TE, PTE, SE | Literature review (local studies) |
| (Ali et al., 2017) | Public & Private Hospitals | 12 | 36 | CCR, BCC | CRS, VRS | Output | No. of medical staffs  No. of beds  Drug costs | No. of outpatients  Inpatient days  No. of surgeries | TE, PTE, SE | Literature review & Data availability |
| (Xenos et al., 2017) | Public General Hospital | 108 | 432 | CCR, BCC | CRS, VRS | Input | FTE doctors  No. of other staffs  No. of actual beds  Total expenditure excluding labour | CaseMix adjusted discharges  No. of diagnostic procedures | TE, PTE, SE | Literature review & Data availability |
| (Flokou et al., 2017a) | Public General Hospital | 107 | 535 | CCR, BCC | CRS, VRS | Input | No. of physicians  No. of medical staffs  No. of beds | No. of outpatient  No. of inpatient  No. of surgeries | TE, PTE, SE | Literature review |
| (Campanella et al., 2017) | Public Hospital | 50 | 50 | CCR | CRS | Input | Ratio doctor:patient admission  Ratio nurse:patient admission  Ratio bed:patient admission | 30-days risk adjusted mortality for acute myocardial infarction  30-days risk adjusted mortality for congestive heart disease  30-days risk adjusted mortality for pneumonia | TE | Literature review & Expert opinion |
| (Anthun et al., 2017) | Public Hospital | 22-55 | 506 | CCR, BCC | CRS, VRS | NA | Operating costs | No. of outpatient visits & treatments  No. of elective inpatient discharges  No. of emergency inpatient discharges | TE, PTE, SE | Literature review |
| (Lacko et al., 2017) | Specialized Hospital | 4 | 24 | CCR, BCC | CRS, VRS | Both | No. of doctors  No. of nurses  No. of beds  Cost per beds | No. of inpatient admission  No. of outpatient visits | TE. PTE | Literature review & Data availability |
| (Klangrahad, 2017) | Tertiary Public Hospital | 24 | 24 | BCC | VRS | Input | No. of beds  Ratio bed:population  Ratio doctor:bed | No. of outpatients  No. of inpatients  No. of emergency visits | PTE | Literature review |
| (Khushalani & Ozcan, 2017) | Public General Hospital | 1259 | 6295 | Dynamic Network | CRS | Both | FTE non-physicians  No. of beds  Cost per beds  Ratio nurse:patient  No. of Hi-tech services | No. of outpatients  CaseMix discharges  No. of emergency visits  No. of surgeries  Revenue per beds  Patient score rating | TE | Literature review |
| (Guerrini et al., 2018) | Public & Private Hospitals | 72 | 142 | CCR | CRS | Input | No. of beds  Operating costs | No. of emergency visits  Inpatient revenues  Clinical examination revenues | TE | Literature review |
| (Zheng et al., 2018) | Public Hospital | 84 | 588 | CCR, BCC | CRS, VRS | Input | No. of staffs  No. of beds  No. of fixed assets  Government financial subsidies | No. of outpatient & emergency visits  No. of discharges  Total revenues  BOR | TE, PTE, SE | Literature review & Bibliometric analysis |
| (Guo et al., 2018) | Public Hospital | 18 | 90 | SBM | NR | Non-oriented | FTE staffs  No. of beds  Inpatient discharge rates | Inpatient days  No. of emergency visits  No. of outpatient  Mortality rate | TE | Literature review, Stakeholder’s opinion & Data availability |
| (Patra & Ray, 2018) | District Hospital | 21 | 21 | BCC | VRS | Output | No. of physicians  No. of medical staffs  No. of beds | No. of outpatients  No. of emergency visits  No. of discharges | PTE | Literature review |
| (Irwandy & Sjaaf, 2018) | Public Hospital | 26 | 26 | BCC | VRS | Output | No. of doctors  No. of nurses  No. of other health workers  No. of non-health workers  No. of beds | No. of outpatients  No. of inpatients  No. of emergency visits  BOR  ALOS | PTE | Literature review & Data availability |
| (Pirani et al., 2018) | University Affiliated Public Hospital | 17 | 85 | BCC | VRS | Output | No. of nurses  No. of actual beds  No. of admission | ALOS  TOI | PTE | Literature review |
| (Zhang et al., 2018) | Public Hospital | 213 | 1278 | SBM | CRS, VRS | Non-oriented | No. of doctors  No. of nurses  No. of other staffs  No. of actual beds  Total expenditure  Hospital building areas | Ratio outpatient:day  Average admission & discharges  No. of emergency beds | TE, PTE | Literature review |
| (Sultan & Crispim, 2018b) | Public Hospital | 11 | 66 | CCR, BCC | CRS, VRS | Input | FTE doctors  FTE medical staffs  FTE administrative staffs  No. of beds | No. of outpatients  CaseMix adjusted inpatients  No. of emergency visits | TE, PTE, SE | Literature review |
| (Sultan & Crispim, 2018a) | Public Hospital | 35 | 198 | CCR, BCC | CRS, VRS | Input | FTE doctors  FTE medical staffs  FTE administrative staffs | No. of outpatients  CaseMix adjusted inpatients  No. of emergency visits without admission | TE, PTE | Literature review |
| (Stefko et al., 2018) | Tertiary Public Hospital | 8 | 56 | CCR, BCC | CRS, VRS | Output | No. of medical staffs  No. of beds  No. of CT machine  No. of MRI machine  No. of medical devices | BOR  ALOS | TE, PTE, SE | Literature review |
| (Ho et al., 2018) | Public & Private Hospitals | 12 | 12 | CCR | CRS | Input | No. of medical staffs  No. of beds | No. of outpatients  No. of inpatients | TE | Literature review & DMU limitation |
| (Hung & Wu, 2018) | Public Hospital | 163 | 163 | CCR | CRS | Input | No. of physicians  No. of nurses  No. of other staffs  No. of acute beds  No. of ICU beds  No. of other beds | No. of CaseMix admission  No. of outpatients  No. of emergency visits  No. of operations  No. of revisit outpatients (within 24-h)  No. of revisit emergency (within 24-h)  No. of revisit emergency (within 3-days) | TE | Literature review |
| (Giménez et al., 2019) | District Hospital | 602 | 3010 | NA | NA | NA | Medical personnel costs  Administrative personnel costs  Drugs cost  Goods & services costs | Weighted production values | NA | Literature review |
| (Y. Li et al., 2019) | Public Hospital | 37 | 155 | BCC | VRS | Input | FTE medical staffs  No. of beds | No. of emergency visits  ALOS  No. of specialist outpatients  No. of general outpatients  No. of family medicine clinics  No. of allied health outpatients | PTE | Literature review |
| (Chen et al., 2019) | Public & Non-profit Hospitals | 119 | 833 | CCR | CRS | NA | No. of physicians  No. of nurses  No. of medical staffs  No. of beds | No. of outpatient & emergency visits  Inpatient days  No. of surgeries | TE | Literature review |
| (Wu & Wu, 2019) | Public & Private Hospitals | 329 | 329 | Robust DEA | NR | NR | FTE nurses  No. of beds  Personnel costs  Supplies costs  Space area per beds  Private room ratio | No. of outpatients  No. or emergency visits  DRG-discharges  Specialization index  Technology index | TE | Literature review |
| (Ahmed et al., 2019) | District Hospital | 62 | 62 | CCR, BCC | CRS, VRS | Input | No. of doctors  No. of nurses  No. of beds | No. of antenatal care services  No. of postnatal care services  No. of normal deliveries  No. of caesarean sections  No. of outpatients  No. of inpatient admissions | TE, PTE, SE | Literature review & Data availability |
| (B. Li et al., 2019) | Tertiary Public Hospital | 29 | 232 | CCR | CRS | Output | No. of medical staffs  No. of beds  Township hospital status | No. of outpatients  No. of inpatients  BOR | TE | Literature review |
| (Jing et al., 2019) | Public & Private Hospitals | 154-232 | 1150 | CCR, BCC | CRS, VRS | Output | No. medical staffs  No. of beds | No. of outpatient & emergency visits  No. of discharges  Total revenues | TE, PTE, SE | Literature review |
| (Franco Miguel et al., 2019) | Public, Private & Mixed Hospitals | 25 | 144 | CCR, BCC | CRS, VRS | Input | FTE doctors  No. of beds  Total expenditure | No. of outpatients  No. of emergency visits  DRG-discharges  No. of daycare surgeries | TE, PTE | Literature review |
| (Cinaroglu, 2019) | Public Hospital | 81 | 81 | BCC | VRS | Input | FTE physicians & general practitioners  FTE nurses & midwives  No. of actual beds | No. of admissions  No. of inpatients  No. of surgeries (inpatient & outpatient) | PTE | Literature review |
| (İlgün & Konca, 2019) | Training & Research Hospitals | 47 | 188 | BCC | VRS | Input | No. of specialists  No. of beds  No. of ICU beds | No. of outpatients  No. of inpatients  No. of surgeries (Type A)  No. of surgeries (Type B)  No. of surgeries (Type C) | PTE | Literature review |
| (Şahin & İlgün, 2019) | Public Hospital | 865 | 2595 | Super, BCC | VRS | Input | No. of doctors  No. of nurses & midwives  No. of other medical staffs  No. of beds | No. of inpatients  No. of clinic admissions  No. of surgeries  Mortality rate | PTE | Literature review |
| (Zhao et al., 2020) | Public Hospital | 31 | 31 | CCR, BCC | CRS, VRS | Input | No. of hospital staffs  No. of beds  No. of hospitals | No. of inpatients  No. of discharges  BOR  ALOS | TE, PTE, SE | Literature review & Data availability |
| (Küçük et al., 2020) | Public Hospital | 669 | 3345 | BCC | VRS | Output | No. of doctors  No. of beds  No. of CT & MRI machines  Total expenditure | No. of outpatients  No. of inpatients  No. of emergency visits  No. of surgeries | PTE | Literature review |
| (Hunt & Link, 2020) | Public General Hospital | 6523 | 6523 | NA | NA | Input | FTE nurses  No. of medical staffs  No. of actual beds | No. of admissions  Inpatient days  No. of discharges (Type A)  No. of discharges (Type B)  No. of emergency visits  No. of surgeries | NA | Literature review |
| (Berger et al., 2020) | Public & Private (Non-profit) Hospitals | 120 | 2530 | Super | VRS | Input | No. of physicians  No. of nurses  No. of other staffs  Imputed costs  Primary costs  Secondary costs  Operating costs  Medical & consumable costs | DRG credits  MEL credits  HDG & others credits | PTE | Literature review |
| (Schneider et al., 2020) | Acute Care Hospital | 1428 | 4094 | BCC | VRS | Input | No. of physicians  No. of nurses  No. of assistant nurses  No. of acute beds | No. of outpatients  CaseMix adjusted discharges | PTE | Literature review |
| (Irwandy et al., 2020) | Public Hospital | 25 | 100 | Super | VRS | Output | Total number of hospital assets  Pharmacy costs  Operating costs  Hospital building areas | No. of patients  No. of surgeries  No. of laboratory examinations  ALOS  TOI  Operating income | PTE | Literature review & Data availability |
| (Asiabar et al., 2020) | Public Hospital | 29 | 116 | BCC | VRS | Input | No. of doctors  No. of nurses  No. of other staffs  No. of beds | No. of outpatients  No. of inpatients  No. of surgeries  BOR | PTE | Literature review & Delphi technique |
| (Jahantigh & Ostovare, 2020) | Teaching Hospital | 40 | 40 | BCC | VRS | Output | No. of doctors  No. of nurses  No. of beds  No. of special beds | No. of outpatients  No. of admissions  No. of emergency visits  No. of admissions (with complications)  No. of admissions (without complications)  Infant mortality rate  Staff satisfaction score  Patient satisfaction score | PTE | Literature review & Promethee method |
| (Alatawi et al., 2020) | Public Hospital | 91 | 91 | CCR, BCC | CRS, VRS | Input | No. of physicians  No. of nurses  No. of allied health staffs  No. of beds | No. of outpatients  No. of discharges  No. of surgical operations  No. of radiology & laboratory tests  Mortality rate | TE, PTE | Literature review |
| (Ortega-Díaz et al., 2020) | Public & Private Hospitals | 230 | 690 | CCR, BCC | CRS, VRS | Input | No. of medical staffs  No. of other medical staffs  No. of other staffs  No. of beds  Outsourcing costs | No. of outpatients  No. of emergency visits  No. of surgeries  CaseMix adjusted discharges | TE, PTE, SE | Literature review |
| (Ghahremanloo et al., 2020) | University Hospital | 11 | 33 | CCR | CRS | Non-oriented | No. of medical staffs  No. of other staffs  No. of beds | BOR  BTR | TE | Literature review & Expert opinion |
| (Ayiko et al., 2020) | Public & Private (Non-profit) Hospitals | 78 | 234 | CCR, BCC | CRS, VRS | Input | No. of medical staffs  No. of beds | No. of outpatients  No. of inpatients  No. of birth deliveries | TE, PTE, SE | Literature review |
| (Gao & Wang, 2021) | Public Hospital | 202 | 202 | BCC | VRS | Input | No. of physicians  No. of nurses  No. of medical staffs  No. of beds | No. of outpatients  No. of discharges | PTE | Literature review (local studies) |
| (Cinaroglu, 2021) | Public Hospital | 628-633 | 2512 | BCC | VRS | Input | FTE physicians & general practitioners  No. of actual beds | No. of inpatients  No. of surgeries (inpatient & outpatient) | PTE | Literature review |
| (Nguyen & Zelenyuk, 2021) | Teaching & Non-teaching Hospitals | 104 | 520 | CCR, BCC | CRS, VRS | Output | FTE medical staffs  No. of beds  Medical, surgical & drugs costs | Price adjusted outpatients  CaseMix adjusted inpatients | TE | Literature review & Data availability |
| (Garmatz et al., 2021) | Teaching Hospital | 29 | 29 | CCR | CRS | Output | FTE doctors  FTE nurses  FTE nursing technicians  No. of beds | Inpatient days  Total admissions  Total revenues  No, of high complex procedures | TE | Literature review |
| (Yin et al., 2021) | Tertiary Public Hospital | 25 | 125 | CCR | CRS | Output | No. of doctors  No. of nurses  No. of assistant doctors  No. of actual beds | No. of outpatient & emergency visits  No of discharges  No. of surgeries  BOR  Mortality rate  No. of medical inquiries | TE | Literature review & Delphi technique |
| (Peng et al., 2021) | All Hospitals | 200 | 200 | BCC | VRS | Input | No. of physicians  No. of nurses  No. of medical staffs  No. of other staffs  No. of beds  Operating costs | No. of outpatients  No. of inpatients  No. of emergency visits  No. of discharges  No. of surgeries  Total revenues  No. of family planning clinics  ALOS | PTE | Literature review |
| (See & Ng, 2021) | Public & Non-profit Hospitals | 62-74 | 492 | GMMPI | NA | NA | No. of doctors  No. of medical staffs  No. of administrative staffs  No. of beds | No. of outpatient & emergency visits | NA | Literature review & Data availability |
| (Vrabková & Vaňková, 2021) | Public Hospital | 47 | 47 | CCR, BCC | CRS, VRS | Input | No. of physicians  No. of nurses  No. of other staffs | No. of outpatients  No. of inpatients  No. of surgeries | TE, PTE, SE | Literature review |
| (Goudarzi et al., 2021) | University & Non-university Hospitals | 19 | 38 | BCC | VRS | Input | No. of physicians  No. of nurses  No. of actual beds | No. of inpatients  BOR | PTE | Literature review |
| (Piubello Orsini et al., 2021) | Public Hospital | 43 | 86 | CCR | CRS | Input | FTE nurses  FTE medical staffs  FTE administrative staffs  FTE technical staffs  Operating costs | No. of outpatients  CaseMix adjusted inpatients  BOR  Inpatient revenues  Outpatient revenues | TE | Literature review |
| (Pereira et al., 2021) | Public Hospital | 27 | 27 | Matrix Network | VRS | NA | Medical staff costs  Medical & consumable costs  Operating costs  Outsourcing costs | No. of inpatients  No. of emergency visits  No. of medical outpatient visits  No. of surgeries  No. of emergency surgeries  No. of minor surgeries | PTE | Literature review & Stakeholder’s opinion |
| (Fumbwe et al., 2021) | Public Hospital | 130 | 130 | BCC | VRS | Input | No. of medical staffs  No. of beds | No. of outpatients  No. of inpatients | PTE | Literature review |
| (Kim et al., 2021) | Acute Care Hospital | 153-178 | 702 | NA | NA | Output | FTE doctors  FTE nurses  No. of beds | No. of outpatients  Inpatient days  No. of surgeries | NA | Literature review |
| (Babalola et al., 2022) | District Hospital | 38 | 114 | CCR, BCC | CRS, VRS | Input | No. of medical& dental staffs  No. of nurses  No. of pharmacists  No. of allied health staffs  No. of supporting and other personnel  No. of beds | No. of outpatient visits  Inpatient days  Number of operating theatres  No. of X-ray  No. of caesarean sections  No. of normal deliveries | TE, PTE, SE | Literature review |
| (Karma & Gashi, 2022) | Public Hospital | 8 | 8 | CCR | CRS | Input | No. of specialist  No. of beds | No. of inpatient discharges  No. of surgical operations | TE | Literature review & DMU limitation |
| (Zhu & Song, 2022) | Tertiary Public Hospital | 10 | 30 | BCC | VRS | Output | No. of total staffs  No. of beds | No. of outpatient & emergency visits  No. of discharges | PTE | Literature review & DMU limitation |
| (Zarrin, 2022) | University Hospital | 28 | 28 | SBM | NR | Non-oriented | FTE physicians  FTE nurses  No. of beds | Adjusted inpatients  No. of outpatients  No. of medical students  No. of graduates  3^rd^ party funding incomes | TE | Literature review & DMU limitation |
| (Zarrin et al., 2022) | All Hospitals | 1100 | 1100 | SBM | VRS | Input | No. of physicians  No. of nurses  No. of beds | No. of outpatients  CaseMix adjusted inpatients  No. of surgeries | PTE | Literature review |
| (Yousefi Nayer et al., 2022) | Public Hospital | 15 | 15 | CCR, BCC | CRS, VRS | Input | No. of hospital staffs  No. of beds | No. of patients  No. of surgeries  ALOS | TE, PTE, SE | Literature review |
| (Dohmen et al., 2022) | Public & Private General Hospitals | 72 | 576 | CCR | CRS | Input | FTE doctors  FTE medical & non-medical staffs  Operating costs | No. of outpatient visits  No. of inpatient admissions  No. of daycare treatments | TE | Literature review |
| (Ortega-Díaz & Martín, 2022) | Public General Hospital | 232 | 232 | SBM | VRS | Input | FTE medical staffs  FTE other staffs  No. of beds  Supplies costs | No. of outpatients  No. of emergency visits  CaseMix adjusted discharges  CaseMix adjusted death | PTE | Literature review |
| (Onder et al., 2022) | Acute Care Hospital | 2997 | 2997 | BCC | VRS | Output | Log of beds number  Log of hospital staff number  Log of discharges number | Negative-log of readmission rate  Negative-log of operating costs | PTE | Literature review |
|  |  |  |  |  |  |  |  |  |  |  |
