# Supplementary material for: Approach in inputs & outputs selection of Data Envelopment Analysis (DEA) efficiency measurement in hospitals: A systematic review
Source: PLoS One. 2024 Aug 14;19(8):e0293694. doi: 10.1371/journal.pone.0293694 (PMC11324144; doi:10.1371/journal.pone.0293694)
Supplement: S4 Appendix — (DOCX) [file pone.0293694.s004.docx]

Appendix D

**Table 7**

Summary of 89 reviewed publications

| **Author(s) (year)** | **Country** | **Data types** | **Study period (years)** | **Number of models** | **Extended analysis** | **Extended analysis methods** |
| --- | --- | --- | --- | --- | --- | --- |
| (Czypionka et al., 2014) | Austria | Cross sectional | 1 | 2 | Yes | Ordinary least square regression  Spearman rank correlation coefficient |
| (Fragkiadakis et al., 2014) | Greece | Panel | 5 | 2 | Yes | Bootstrap  Truncated regression  Malmquist productivity index |
| (Özgen Narcı et al., 2014) | Turkey | Cross sectional | 1 | 2 | Yes | Tobit regression |
| (Cavalieri et al., 2014) | Italy | Cross sectional | 12 | 4 | Yes | Bootstrap  Ordinary least square regression  Truncated regression  Mann-Whitney test  Pearson correlation coefficient |
| (Matranga & Sapienza, 2015) | Italy | Cross sectional | 1 | 1 | Yes | Bootstrap  Kruskal-Wallis test |
| (Gok & Altındağ, 2015) | Turkey | Cross sectional | 8 | 1 | Yes | Spearman rank correlation coefficient |
| (H. Li & Dong, 2015) | China | Cross sectional | 1 | 1 | Yes | Bootstrap  Peers ranking |
| (Cheng et al., 2015) | China | Panel | 3 | 1 | Yes | Tobit regression  Malmquist productivity index |
| (Tiwari & Shukla, 2015) | India | Cross sectional | 2 | 1 | No |  |
| (Rezaee & Karimdadi, 2015) | Iran | Cross sectional | NA | 1 | Yes | Cluster analysis |
| (Almeida et al., 2015) | Portugal | Cross sectional | 1 | 2 | Yes | Wilcoxon matched-pairs signed-rank test |
| (Chowdhury & Zelenyuk, 2016) | Canada | Panel | 2 | 7 | Yes | Bootstrap  Truncated regression  Li test statistic |
| (van Ineveld et al., 2016) | Dutch | Cross sectional | 2 | 2 | Yes | Malmquist productivity index |
| (Villalobos-Cid et al., 2016) | Chile | Cross sectional | 1 | 1 | Yes | MST-kNN algorithm cluster analysis  Kruskal-Wallis test  K-means cluster analysis  Isodata cluster analysis |
| (Wang et al., 2016) | China | Cross sectional | 1 | 1 | Yes | Tobit regression |
| (Str & Kalogeropoulou, 2016) | Greece | Cross sectional | 2 | 1 | Yes | Bootstrap  Spearman rank correlation coefficient |
| (ArulJothi, K et al., 2016) | India | Cross sectional | 1 | 1 | Yes | Peers ranking |
| (R. C. Li et al., 2016) | Philippines | Cross sectional | NA | 1 | No |  |
| (Caballer-Tarazona et al., 2016) | Spain | Cross sectional | 2 | 1 | Yes | Linear regression  Cluster analysis |
| (Mujasi et al., 2016) | Uganda | Cross sectional | 1 | 1 | Yes | Tobit regression |
| (Flokou et al., 2017b) | Greece | Cross sectional | 1 | 1 | Yes | Bootstrap |
| (Valdmanis et al., 2017) | Scotland | Cross sectional | 5 | 1 | Yes | Bootstrap  Ordinary least square regression  Malmquist productivity index |
| (Soares et al., 2017) | Brazil | Cross sectional | 1 | 1 | Yes | Peers ranking |
| (Jiang et al., 2017) | China | Panel | 7 | 1 | Yes | Tobit regression |
| (Ali et al., 2017) | Ethiopia | Panel | 2 | 1 | Yes | Tobit regression |
| (Xenos et al., 2017) | Greece | Panel | 4 | 3 | Yes | Bootstrap  Tobit regression  Malmquist productivity index |
| (Flokou et al., 2017a) | Greece | Cross sectional | 4 | 1 | Yes | Window analysis  Malmquist productivity index |
| (Campanella et al., 2017) | Italy | Cross sectional | 1 | 1 | Yes | Bootstrap  Tobit regression  Multivariate & univariate analysis |
| (Anthun et al., 2017) | Norway | Cross sectional | 16 | 1 | Yes | Bootstrap  Malmquist productivity index |
| (Lacko et al., 2017) | Slovakia | Panel | 6 | 1 | Yes | Bootstrap |
| (Klangrahad, 2017) | Thailand | Cross sectional | 1 | 1 | Yes | Peers ranking |
| (Khushalani & Ozcan, 2017) | USA | Cross sectional | 5 | 1 | Yes | Multinomial logit regression  Unpaired t-test  Malmquist productivity index |
| (Guerrini et al., 2018) | Italy | Cross sectional | 2 | 1 | Yes | Tobit regression  Ordinary least square regression  Stochastic Frontier analysis  Mann-Whitney test |
| (Zheng et al., 2018) | China | Panel | 7 | 1 | Yes | Tobit regression  Fitting adjustment |
| (Guo et al., 2018) | Hong Kong | Panel | 2 | 1 | Yes | Tobit regression |
| (Patra & Ray, 2018) | India | Cross sectional | 1 | 1 | Yes | Ordinary least square regression |
| (Irwandy & Sjaaf, 2018) | Indonesia | Cross sectional | 1 | 1 | No |  |
| (Pirani et al., 2018) | Iran | Cross sectional | 5 | 1 | Yes | Beta regression |
| (Zhang et al., 2018) | Japan | Cross sectional | 6 | 1 | Yes | Malmquist productivity index |
| (Sultan & Crispim, 2018b) | Palestine | Cross sectional | 6 | 6 | Yes | Tobit regression  Window analysis |
| (Sultan & Crispim, 2018a) | Palestine | Cross sectional | 6 | 1 | No |  |
| (Stefko et al., 2018) | Slovakia | Cross sectional | 7 | 8 | Yes | Window analysis |
| (Ho et al., 2018) | Taiwan | Cross sectional | 1 | 1 | No |  |
| (Hung & Wu, 2018) | Taiwan | Cross sectional | 1 | 1 | Yes | Kruskal-Wallis test |
| (Giménez et al., 2019) | Colombia | Cross sectional | 5 | 1 | Yes | Global Malmquist-Luenberger index |
| (Y. Li et al., 2019) | Hong Kong | Cross sectional | 2 | 1 | Yes | Spearman rank correlation coefficient |
| (Chen et al., 2019) | Taiwan | Cross sectional | 7 | 1 | Yes | Malmquist productivity index |
| (Wu & Wu, 2019) | USA | Cross sectional | 1 | 6 | No |  |
| (Ahmed et al., 2019) | Bangladesh | Cross sectional | 1 | 1 | Yes | Tobit regression |
| (B. Li et al., 2019) | China | Cross sectional | 4 | 1 | Yes | Theil index  Grey correlation  Malmquist productivity index |
| (Jing et al., 2019) | China | Cross sectional | 6 | 1 | Yes | Tobit regression  Propensity score matching |
| (Franco Miguel et al., 2019) | Spain | Cross sectional | 6 | 4 | Yes | Bootstrap  Malmquist productivity index |
| (Cinaroglu, 2019) | Turkey | Cross sectional | 1 | 1 | Yes | K-means cluster analysis  F-test |
| (İlgün & Konca, 2019) | Turkey | Cross sectional | 4 | 1 | Yes | Spearman rank correlation coefficient |
| (Şahin & İlgün, 2019) | Turkey | Cross sectional | 6 | 1 | Yes | Wilcoxon signed-rank test  Malmquist productivity index |
| (Zhao et al., 2020) | China | Panel | 5 | 1 | Yes | Malmquist productivity index |
| (Küçük et al., 2020) | Turkey | Cross sectional | 5 | 1 | No |  |
| (Hunt & Link, 2020) | USA | Cross sectional | 3 | 1 | Yes | Bootstrap  Tobit regression  Ordinary least square regression |
| (Berger et al., 2020) | Austria | Cross sectional | 14 | 3 | Yes | Ordinary least square regression  Bias corrected bootstrap  F-test |
| (Schneider et al., 2020) | German | Cross sectional | 3 | 3 | Yes | Bootstrap  Truncated regression |
| (Irwandy et al., 2020) | Indonesia | Cross sectional | 4 | 1 | Yes | Peers ranking |
| (Asiabar et al., 2020) | Iran | Panel | 5 | 2 | Yes | Tobit regression  Repeated measure ANOVA |
| (Jahantigh & Ostovare, 2020) | Iran | Cross sectional | NA | 1 | Yes | Peers ranking |
| (Alatawi et al., 2020) | Saudi Arabia | Cross sectional | 1 | 1 | Yes | Tobit regression  Spearman rank correlation coefficient |
| (Ortega-Díaz et al., 2020) | Spain | Cross sectional | 3 | 1 | Yes | Generalized linear mixed model |
| (Ghahremanloo et al., 2020) | Tehran | Panel | 1 | 2 | No |  |
| (Ayiko et al., 2020) | Uganda | Cross sectional | 3 | 1 | Yes | Tobit regression |
| (Gao & Wang, 2021) | China | Cross sectional | 1 | 1 | Yes | Ordinary least square regression |
| (Cinaroglu, 2021) | Turkey | Cross sectional | 4 | 1 | Yes | Bootstrap  Propensity score matching  Pearson Chi-square test  Spearman rank correlation coefficient  Mann-Whitney test |
| (Nguyen & Zelenyuk, 2021) | Australia | Panel | 5 | 1 | Yes | Bootstrap  Central limit theorem  T-test  Kruskal-Wallis test |
| (Garmatz et al., 2021) | Brazil | Cross sectional | 1 | 1 | Yes | Peers ranking |
| (Yin et al., 2021) | China | Panel | 5 | 3 | Yes | Bootstrap  Kruskal-Wallis test  Malmquist productivity index |
| (Peng et al., 2021) | China | Cross sectional | 1 | 2 | Yes | Tobit regression  Propensity score matching  Ordinary least square regression  T-test |
| (See & Ng, 2021) | China | Panel | 9 | 1 | Yes | Global metafrontier Malmquist productivity index  Ordinary least square regression |
| (Vrabková & Vaňková, 2021) | Czech Republic | Cross sectional | 1 | 4 | Yes | Bootstrap |
| (Goudarzi et al., 2021) | Iran | Cross sectional | 4 | 1 | Yes | Paired T-test  Wilcoxon matched-pairs signed-rank test  Malmquist productivity index |
| (Piubello Orsini et al., 2021) | Italy | Cross sectional | 2 | 1 | Yes | Tobit regression  Malmquist productivity index |
| (Pereira et al., 2021) | Portugal | Cross sectional | 1 | 1 | Yes | Monte Carlo method |
| (Fumbwe et al., 2021) | Tanzania | Cross sectional | 1 | 1 | No |  |
| (Kim et al., 2021) | USA | Cross sectional | 9 | 1 | Yes | Bootstrap  Tobit regression |
| (Babalola et al., 2022) | Africa | Cross sectional | 3 | 1 | Yes | Tobit regression |
| (Karma & Gashi, 2022) | Bosnia | Panel | 3 | 4 | Yes | Tobit regression  Mann-Whitney test |
| (Zhu & Song, 2022) | China | Panel | 3 | 1 | Yes | Pabon-Lasso model |
| (Zarrin, 2022) | German | Cross sectional | 1 | 8 | Yes | Tobit regression |
| (Zarrin et al., 2022) | German | Cross sectional | 1 | 1 | Yes | Bootstrap  Self-organizing map artificial neural network cluster analysis  Mann-Whitney test |
| (Yousefi Nayer et al., 2022) | Iran | Cross sectional | 1 | 1 | Yes | Tobit regression |
| (Dohmen et al., 2022) | Netherlands | Cross sectional | 8 | 8 | Yes | Logistic regression  Ordinary least square regression  Pearson correlation coefficient  Stochastic Frontier analysis  Malmquist productivity index |
| (Ortega-Díaz & Martín, 2022) | Spain | Cross sectional | 1 | 1 | Yes | Wilcoxon signed-rank test  Spearman rank correlation coefficient |
| (Onder et al., 2022) | USA | Cross sectional | 1 | 1 | Yes | Bootstrap  Truncated regression |
|  |  |  |  |  |  |  |
