# Supplementary material for: Approach in inputs & outputs selection of Data Envelopment Analysis (DEA) efficiency measurement in hospitals: A systematic review
Source: PLoS One. 2024 Aug 14;19(8):e0293694. doi: 10.1371/journal.pone.0293694 (PMC11324144; doi:10.1371/journal.pone.0293694)
Supplement: S5 Appendix — (DOCX) [file pone.0293694.s005.docx]

Appendix E

**Table 8**

Types of efficiency studied

| **Types of efficiency** | **N** | **Percentage (%)** |
| --- | --- | --- |
| Pure Technical Efficiency (PTE) | 29 | 32.58 |
| Technical Efficiency (TE) | 26 | 29.21 |
| Technical Efficiency, Pure Technical Efficiency & Scale of Efficiency (SE) | 24 | 26.97 |
| Technical Efficiency & Pure Technical Efficiency | 5 | 5.62 |
| Not stated | 5 | 5.62 |
