# Supplementary material for: Approach in inputs & outputs selection of Data Envelopment Analysis (DEA) efficiency measurement in hospitals: A systematic review
Source: PLoS One. 2024 Aug 14;19(8):e0293694. doi: 10.1371/journal.pone.0293694 (PMC11324144; doi:10.1371/journal.pone.0293694)
Supplement: S6 Appendix — (DOCX) [file pone.0293694.s006.docx]

Appendix F

**Table 9**

Model types applied in the studies

| **Model types** | **N** | **Percentage (%)** |
| --- | --- | --- |
| Radial model |  |  |
| Charnes, Cooper and Rhodes (CCR) & Banker, Chames and Cooper (BCC) | 30 | 33.71 |
| Banker, Chames and Cooper (BCC) | 25 | 28.09 |
| Charnes, Cooper and Rhodes (CCR) | 17 | 19.10 |
|  |  |  |
| Non-Radial and Oriented |  |  |
| Slacks-Based Measure (SBM) | 2 | 2.25 |
| Super Efficiency | 1 | 1.12 |
| Dynamic Network | 1 | 1.12 |
| Matrix Network | 1 | 1.12 |
| Additive | 1 | 1.12 |
| Generalized Metafrontier Malmquist Productivity Index (GMMPI) | 1 | 1.12 |
|  |  |  |
| Non-Radial and Non-Oriented |  |  |
| Slacks-Based Measure (SBM) (non-oriented) | 4 | 4.49 |
|  |  |  |
| Radial and Non-Radial |  |  |
| Slacks-Based Measure (SBM), Charnes, Cooper and Rhodes (CCR) & Banker, Chames and Cooper (BCC) | 1 | 1.12 |
|  |  |  |
| Not stated | 4 | 4.49 |
|  |  |  |
