# Supplementary material for: Approach in inputs & outputs selection of Data Envelopment Analysis (DEA) efficiency measurement in hospitals: A systematic review
Source: PLoS One. 2024 Aug 14;19(8):e0293694. doi: 10.1371/journal.pone.0293694 (PMC11324144; doi:10.1371/journal.pone.0293694)
Supplement: S7 Appendix — (DOCX) [file pone.0293694.s007.docx]

Appendix G

**Table 10**

Model orientation applied in the studies

| **Model orientations** | **N** | **Percentage (%)** |
| --- | --- | --- |
| Input oriented | 49 | 55.06 |
| Output oriented | 23 | 25.84 |
| Non-oriented | 5 | 5.62 |
| Input & Output oriented | 3 | 3.37 |
| Not stated | 9 | 10.01 |
