# Supplementary material for: Approach in inputs & outputs selection of Data Envelopment Analysis (DEA) efficiency measurement in hospitals: A systematic review
Source: PLoS One. 2024 Aug 14;19(8):e0293694. doi: 10.1371/journal.pone.0293694 (PMC11324144; doi:10.1371/journal.pone.0293694)
Supplement: S8 Appendix — (DOCX) [file pone.0293694.s008.docx]

Appendix H

**Table 11**

Return to scale assumption applied in the studies

| **Return to scale assumption** | **N** | **Percentage (%)** |
| --- | --- | --- |
| Constant Return to Scale (CRS) & Variable Return to Scale (VRS) | 32 | 35.96 |
| Variable Return to Scale (VRS) | 29 | 32.58 |
| Constant Return to Scale (CRS) | 18 | 20.22 |
| Nor stated | 10 | 11.24 |
