# Supplementary material for: Approach in inputs & outputs selection of Data Envelopment Analysis (DEA) efficiency measurement in hospitals: A systematic review
Source: PLoS One. 2024 Aug 14;19(8):e0293694. doi: 10.1371/journal.pone.0293694 (PMC11324144; doi:10.1371/journal.pone.0293694)
Supplement: Attachment — Submitted filename: Response to Reviewers.docx [file pone.0293694.s009.docx]

# Response to Reviewers:

André Ramalho, PhD

Academic Editor

PLOS ONE

San Francisco, California, USA

1^st^ May 2024

Dear Dr. André Ramalho:

Thank you for inviting us to submit a revised draft of our manuscript entitled, *"Approach in Inputs & Outputs Selection of Data Envelopment Analysis (DEA) Efficiency Measurement in Hospital: A*

*Systematic Review"* to PLOS ONE. We also appreciate the time and effort you and each of the reviewer have dedicated to providing insightful feedback on ways to strengthen our paper. Thus, it is with great pleasure that we resubmit our article for further consideration. We have incorporated changes that reflect the detailed suggestions you have graciously provided. We also hope that our edits and the responses we provide below satisfactorily address all the issues and concerns you and the reviewer have noted.

To facilitate your review of our revisions, the following is a point-by-point response to the questions and comments delivered in your letter dated 5^th^ Feb 2024 [PONE-D-23-33407]. The responses to the concerns raised by editor and reviewer are below and are color coded as follows: a) Comments from editors or reviewers are shown as *[text]*; b) Our responses are shown as [text].

The comments were very helpful overall, and we are appreciative of such constructive feedback on our original submission. After addressing the issues raised, we feel the quality of the paper is much improved.

**EDITOR SUGGESTIONS:**

1. *[We appreciate the opportunity to consider your work for publication. Your article has undergone a peer review process, and I have also conducted a thorough evaluation. After careful consideration, we have concluded that the manuscript requires major revisions before it can be considered for publication. This decision reflects a consensus between the external reviewer's insights and my own assessment as the second reviewer. The key areas that necessitate revision are as follows:]*
   - **RESPONSE:** [We wish to thank you and the reviewer for the assessment and consideration for publication. We agree with you and did major revisions throughout our paper. In our revisions, we have attempted to address key areas that necessitate revision. Details of the changes is the document “Revised Manuscript with Track Changes”. However, we have retained the structure of the paper to cover broad reach of audience including those who are new to Data Envelopment Analysis]
2. *[Language and Clarity: There are several instances of* *typographical errors and language issues that need to be addressed. For example, in Section 3.2.3, the term "return to scale assumption" should be corrected to "returns to scale assumption". We recommend a thorough and careful revision of the English language used throughout the paper to ensure clarity and accuracy.]*

- **RESPONSE:** [We thank Editor for this comment. We regret for the typographical errors and language issues. Accordingly, we have revised, edited and proofread the whole paper to improve the clarity and accuracy. We have also replaced the pointed-out term and others to be more in line with your comments. We hope that the edited paper clarifies this comment]

3. *[Comparative Analysis from Managerial and Economic Viewpoints: The manuscript currently lacks a comparison of the reviewed papers from managerial and economic perspectives. Such an analysis is crucial for a comprehensive understanding of the subject matter. We advise you to incorporate a detailed comparative analysis that reflects these perspectives, thereby enriching the depth and relevance of your review. Please note that these revisions are substantial and critical to enhancing the quality and scholarly value of your paper.]*
   - **RESPONSE:** [We appreciate your insightful and fair comments. We agreed that the paper lacks in the areas mentioned. However, we believed that the paper should interest broad reach of audience both in and outside of DEA discipline. This includes student and those who a new to DEA. Therefore, we incorporated the managerial and economic analysis by adding crucial statements and specific section to reflects the perspective] [Pages and lines referred to manuscript with All Markup] [ The added analysis is highlighted in yellow in the revised manuscript.]
     (i) P.15, lines 353-354 – We elaborated the checklist used was specific to assess from economic perspective
     (ii) P.36-37, lines 810-815 – We explained the method mentioned acceptable for academic or research purpose. But as manager or economic purpose the method need to be use wisely.
     (iii) P.38, lines 863-865 – We added the statement that, the method stated can formalized the judgmental process. This is important from managerial or economic perspective as they need a concrete base or definitive stand in their decision.
     (iv) P.41, lines 937-943 – We elaborated the motivation of manager or economist are different. This motivation in managerial and economic area will change their judgement.
   - [We added a specific section to enrich the paper as suggested and at the same time keep the paper structure to attract more reader especially those who are new to DEA. P.42-45, lines 951-1005, point 4.2 – Specific section to the main objective of the paper. We expanded the discussion from managerial and economic standpoints and proposed specific recommendation. Again, although this recommendation reflects to the specified group it is also relevance to others who is interested.]
   - [We hope these revisions provide a more balanced and thorough discussion in the paper.]

**REVIEWER 1 COMMENTS:**

4. *[Thank you for giving me the opportunity to review the paper entitled "Approach in Inputs & Outputs Selection of Data Envelopment Analysis (DEA) Efficiency Measurement in Hospital: A Systematic Review" (with Manuscript Number: PONE-D-23-33407).*

*I have completed reading this paper. It is a interesting review paper. However, based on the following comments, I believe the current version of this paper is not appropriate for publication in "PLOS ONE".*

*Thus, I recommend major revision for this paper]*

- **RESPONSE:** [We wish to thank you for your insightful comments and pleased to find that this paper interest you. We revised the paper as you suggested in the document “Revised Manuscript with Track Changes”. Your comments and suggestion have greatly helped us to improve the quality of our paper.]

5. *[There are several dictations errors in the text. For instance, in the title of Section 3.2.3, the term "return to scale assumption" must be modified to the term "returns to scale assumption". English language of this paper must be revised carefully.]*
   - **RESPONSE:** [We thank you for noting this. We are very sorry for this dictations error and for the English language was somewhat inadequate. Thus, we reviewed back the paper and improved in term of language, structure and related term on DEA. Proofreading was done accordingly to make the paper better. We hope the revised paper addressed your concern]
6. *[The authors have not compared the papers reviewed in this paper from managerial and economic viewpoints.]*
   - **RESPONSE:** [Thank you for providing these insights. We agreed the paper lacks from the viewpoints mentioned. We also believed it is more appropriate for the paper to be more general as to attract larger and broader audience. Thus, we decided to incorporated the managerial and economic analysis by adding crucial statements and specific section to reflects the viewpoints. Our response is the same as what we replied to the Editor.] [Pages and lines referred to manuscript with All Markup] [ The added analysis is highlighted in yellow in the revised manuscript.]
     (i) P.15, lines 353-354 – We elaborated the checklist used was specific to assess from economic perspective
     (ii) P.36-37, lines 810-815 – We explained the method mentioned acceptable for academic or research purpose. But as manager or economic purpose the method need to be use wisely.
     (iii) P.38, lines 863-865 – We added the statement that, the method stated can formalized the judgmental process. This is important from managerial or economic perspective as they need a concrete base or definitive stand in their decision.
     (iv) P.41, lines 937-943 – We elaborated the motivation of manager or economist are different. This motivation in managerial and economic area will change their judgement.
   - [We added a specific section to enrich the paper as suggested and at the same time keep the paper structure to attract more reader especially those who are new to DEA. P.42-45, lines 951-1005, point 4.2 – Specific section to the main objective of the paper. We expanded the discussion from managerial and economic standpoints and proposed specific recommendation. Again, although this recommendation reflects to the specified group it is also relevance to others who is interested.]
   - [We hope that these revisions are sufficient to make our paper suitable for publication.]

**CONCLUDING REMARKS**: Thank you for providing us the opportunity to improve our manuscript with your useful suggestions and questions. We worked hard to incorporate your feedback and hope that these changes would persuade you to accept our submission.

Sincerely,

[A Azimatun Noor]

Corresponding Author:

A Azimatun Noor

Associate Prof Dr

Universiti Kebangsaan Malaysia

[Faculty of Medicine UKM

Level 2, Kompleks Pendidikan Perubatan Canselor Tuanku Jaafar

Jalan Yaacob Latif, Bandar Tun Razak

56000 Cheras, Kuala Lumpur

MALAYSIA]

[azimatunnoor@ppukm.ukm.edu.my]

[Tel: +603 – 9145 8693]

[Fax: +603 – 9145 8401]

Additional Contact:

M Zulfakhar Zubir

Dr

Institution/Affiliation Name

[Medical Development Division, Ministry of Health Malaysia, Putrajaya, MALAYSIA]

[dr.zulfakhar@moh.gov.my]

[Tel: +6019 – 6686415]
