# Supplementary material for: Approach in inputs & outputs selection of Data Envelopment Analysis (DEA) efficiency measurement in hospitals: A systematic review
Source: PLoS One. 2024 Aug 14;19(8):e0293694. doi: 10.1371/journal.pone.0293694 (PMC11324144; doi:10.1371/journal.pone.0293694)
Supplement: Attachment — Submitted filename: Response to Reviewers.docx [file pone.0293694.s010.docx]

# Response to Reviewers:

André Ramalho, PhD

Academic Editor

PLOS ONE

San Francisco, California, USA

5^th^ June 2024

Dear Dr. André Ramalho:

Thank you for inviting us to submit a revised version of our manuscript entitled, *"Approach in Inputs & Outputs Selection of Data Envelopment Analysis (DEA) Efficiency Measurement in Hospital: A Systematic Review"* to PLOS ONE. We also appreciate the time and effort you and each of the reviewers have dedicated to providing insightful feedback on ways to strengthen our paper. Thus, it is with great pleasure that we resubmit our article for further consideration. We have incorporated changes that reflect the detailed suggestions you have graciously provided. We also hope that our edits and the responses we provide below satisfactorily address all the issues and concerns you and the reviewer have noted.

To facilitate your review of our revisions, the following is a point-by-point response to the questions and comments delivered in your letter dated 21^st^ May 2024 [PONE-D-23-33407R1]. The responses to the concerns raised by editor and reviewers are below and are color coded as follows: a) Comments from editors or reviewers are shown as *[text]*; b) Our responses are shown as [text].

The comments were very helpful overall, and we are appreciative of such constructive feedback on our original submission. After addressing the issues raised, we feel the quality of the paper is much improved.

**EDITOR SUGGESTIONS:**

1. *[Thank you for submitting your manuscript to PLOS ONE. After careful consideration, we feel that it has merit but does not fully meet PLOS ONE’s publication criteria as it currently stands. Therefore, we invite you to submit a revised version of the manuscript that addresses the points raised during the review process. Minor Revision]*
   - **RESPONSE:** [We are really glad that the article reaches you. We also appreciate the time and effort you and each of the reviewer have dedicated to providing insightful feedback on ways to strengthen our paper. Previously the article needs a major revision, we are delightful that the second revision require minor revision. Thus, it is with great pleasure that we resubmit our article for further consideration. We have incorporated changes that reflect the detailed suggestions you have graciously provided. We also hope that our edits and the responses we provide below satisfactorily address all the issues and concerns you and the reviewers have noted.]
   - [We appreciate both of the reviewers insightful and fair comments. Thank you for Reviewer #1 for the recommendation and acceptance for publication. We agreed with Reviewer #2 for the additional comment and suggestion. Therefore, we incorporated the concerns raised by Reviewer #2] [Pages and lines referred to manuscript with All Markup] [ The added content or response to comment is highlighted in yellow in the revised manuscript.]
   - [These changes, we hope, will provide the paper a more comprehensive and fair discussion.]

**REVIEWER 1 COMMENTS:**

*[Dear Editor,*

*Thank you for giving me the opportunity to review the revised paper entitled "Approach in Inputs & Outputs Selection of Data Envelopment Analysis (DEA) Efficiency Measurement in Hospitals: A Systematic Review" (with Manuscript Number: PONE-D-23-33407R1).*

*I have completed reading the revised paper. In my opinion, the authors have addressed the points raised in the previous review with patient.*

*I believe that the revised paper is now acceptable for publication in "PLOS ONE".*

*Yours sincerely,]*

- **RESPONSE:** [We would like to thank you for taking the necessary time and effort to review the article. We sincerely appreciate all your valuable comments and suggestions, which helped us in improving the quality of the article. We would like to take this opportunity to thank you for the effort and expertise that you contributed towards reviewing the article, without which it would be impossible to maintain the high standards of peer-reviewed journals. Thank you for considering the article acceptable for publication in “PLOS ONE”.]

**REVIEWER 2 COMMENTS:**

1. *[Thank you for the opportunity of reviewing this work. Authors conducted a systematic review on the use of DEA for performing hospital efficiency assessment, focusing on input-output selection. This review touches a very relevant topic in health economics. Authors have assessed a considered number of publications, summarizing and describing key methodological aspects behind the use of DEA for hospital efficiency assessment. Overall, the issue is relevant, and the review is worth for publication, but some major improvements need to be done. Below I provide comments and suggestions separated by sections:]*

- **RESPONSE:** [We wish to thank you for your insightful comments and pleased to find that this paper interest you. We revised the paper as you suggested in the document “Revised Manuscript with Track Changes”. Your comments and suggestion have greatly helped us to improve the quality of our paper.]
- [ The added content or response to comment is highlighted in yellow in the revised manuscript.]
- [We hope these revisions provide a more balanced and thorough discussion in the paper.]

2. *[Introduction
   Line 66: "[...] and a framework is designed. in keeping with the analysis. [...]" -> It seems there is text missing in this sentence.*

*Line 133: " [...] Despite the large number of studies on the use of DEA in hospitals, efforts to comprehensively examine these studies are still missing and warrant more investigation. This article identifies and describes DEA research in hospitals in an effort to close the knowledge gap. [...]" -> I am afraid this may not be entirely true. There are several published review articles that have assessed and described the use of DEA in the hospital sector, including all methodological aspects within this methodology, such as input-output selection. Having said that, the motivation and need for this review should be clarified and authors need to clearly state what the review adds to other existing and similar reviews.*

*Line 143: "In addition, this study observed the current trend in analysing hospital efficiency using DEA" -> What does this objective means exactly? It does not seem that this analysis was performed in the Results sections. Could you please clarify this point?]*

- **RESPONSE:** [We thank you for noting this. Some of the comments were already changed in the first review. We will point out the comment based on the line you mentioned.]
- Line 66: This statement was changed in the manuscript.
  - Line 66: A framework is required following the analysis process. The optimal approach to implementing performance measurement is not to identify a minor adjustment as a supporting role to enhance one aspect of the health system outcomes. Instead, this identification should be utilised as a general strategy in gauging performance among the various system components.
- Line 133: This statement was changed to reflect and clarify the motivation for the produce manuscript. We thank you for mentioning this.
  - Line 125: Although numerous hospital-based DEA articles have been recorded, inadequate complete analysis has been observed. Consequently, this outcome requires further investigation, leading to a research gap involving hospital-based DEA articles.
- Line 143: We revised this statement to explain that the review addressed the research gap mentioned. What we meant by the current trend is that we looked at few findings in previous review and compare briefly in the Discussion section.
  - Line 131: To the authors' knowledge, no reviews regarding hospital-based DEA articles involving optimal input and output variable selections were reported. Thus, this review addressed this research gap by observing the current trends in hospital-based DEA analyses.
  - Line 636: Consequently, these findings align with other healthcare-based reviews [17,21,33].
  - Line 651: Previous articles also highlighted similar findings with varying proportions [17,20,21].
  - Line 667: Previous articles also demonstrated a trend towards replacing CRS with VRS assumption in DEA-based applications [20,21,33].
  - Line 681: Consequently, this finding was similar to previous DEA-related and performance-based articles on healthcare services [180,181].

3. *[Methods
   Authors used 6 standard, comprehensive databases and justified their choice. However, Methods section lacks description on the search strategy and query. It was only mentioned the terms used. It is important to present at least the final query used for searching the databases. Also, it is not completely clear how many reviewers participated in each phase (abstract screening, full-text analysis and data extraction). Authors only mention 2 readers and then a third one for consensus in the Quality Appraisal section. Authors need to clarify how the review process was carried out. Furthermore, inclusion and exclusion criteria considered for abstract screening and full-text selection are not explicitly mentioned in the Methods section. This information is usually basic in systematic reviews. Authors only mentioned type of publication, language and period criteria.]*
   - **RESPONSE:** [Thank you for providing these insights. We agreed the paper lacks from the viewpoints mentioned. Some of the comments were already addressed in the first review.]
   - We added the final query for the searching database. General query string is added as the six databases have different search setting and requirement.
   - We added in information on reviewers and method involve in each phase.
     i. Line 179: The records were exported from the databases into Microsoft Excel sheet for screening. The final query string is as follow:
     ii. Line 186: The titles and abstracts were independently screened by three reviewers.
     iii. Line 228: Four reviewers extracted the data independently using a standardized data extraction form which is organized using Microsoft Excel.
   - The review process was also explained in the Quality Appraisal section
     i. Line 216: Thus, the scientific soundness of the chosen article was investigated using two tools to improve robustness and minimise bias. Two co-authors from different institutions evaluated each selected article separately using both tools to enhance reliability. A third reviewer was then requested to assess an article if a disagreement occurred.
     ii. We believe this statement is adequate in explaining the process
   - The inclusion and exclusion criteria were mentioned in the manuscript and in the PRISMA diagram.
     i. Line 187 to 191 explained in details with regards to this matter.
     ii. Line 194: PRISMA diagram further explained the process for final articles to be included in the review.
     iii. We believe this will give better flow to audience in reading the manuscript.
4. *[Results
   I think Tables 1 and 2 need to be restructured. It would be easier to read if Table 1 contains only the full list of input variables and Table 2 only the full list of output variables (including categories and counts), for instance. Moreover, it is important to include the definitions and units of the variables in these tables.*

*The section* *"3.2.5 Extended analysis & data type" only lists a series of additional methods employed but provide little to no information on the purpose or usefulness of applying these extended analyses. For instance, some of the methods presented are typically employed in second-stage analysis (e.g., Tobit regression) to explain the efficiency or inefficiency levels found by DEA. Without this information, I do not see what this section adds.]*

- **RESPONSE:** [Thank you and we appreciate on the suggestion for the table structure. Table 1 are summary for both input and output and it is presented in aggregated information. We believe this is appropriate for those who are well verse in DEA for quick summary. While Table 2 and 3 are details that explained Table 1. The table 2 and 3 are in raw data which include the self-explained definitions and units.]
  - For example, Table 1 for “Beds” is general term, Table 2 describe the type of beds, and units of measurement.
- Thank you for providing these insights. We agreed the paper lacks from the viewpoints mentioned "3.2.5 Extended analysis & data type". Thus, we elaborated this area in the discussion and at the same time we have mentioned the important of this section briefly in previous revision. The added content or response to comment is highlighted in yellow in the revised manuscript.
  - Line 76: Although the theoretical and methodological limitations have been acknowledged in DEA, this method has attracted interest from researchers who aim to address the limitations. Hence, these studies have developed multiple methods integrating DEA with other statistical techniques and methodologies to improve efficiency evaluation.
  - Line 121: and development of novel knowledge and approaches concerning DEA assessment.
  - Line 284: This model could be further analysed or extended through a second stage or integrated with other statistical methods. Consequently, this process could improve efficiency measurement, understanding of the variation or difference in organisational performance, and evaluation of the productivity of the organisation over a specific period.
  - Line 697 – 717: New section “4.2.5 Extended Analysis”

5. *[Discussion and conclusions*

*Authors provided an in-depth discussion on input-output selection, highlighting the existing variability concerning the selection of inputs and outputs. Based on the presented results, it seems that input-output selection is mostly influenced by data availability and research scope, and thus it is not a methodological preference, making it difficult to suggest common practices. However, I missed the main message to be conveyed by the review. I was expecting to find more concrete recommendations and guidelines regarding input-output selection in the Discussion and Conclusion sections, or at least a more directed discussion on how variable selection affects efficiency estimation or what key considerations for variable selection should be addressed.*

*Finally, it is important that authors carefully review the text in order to improve clarity and English.]*

- **RESPONSE:** [We appreciate the comment on this. We added this section during the first revision. Section “4.2 Managerial and economic implications in the input and output selection processes” explained in detail with regards to the review. We outlined the recommendations and guidelines in input-output selection.]
- Furthermore, we also remark few statements in the review to reflects on these perspectives
  - Line 208: This checklist assessed the article from an economic perspective to ensure the findings could be used in policy analysis and managerial decisions.
  - Line 509: Even though this method remained valid for academicians, other assessors (managers, economists or policymakers) could perceive it as contradictory to their practical perspectives. Hence, several factors must be considered from these individuals’ perspectives, including different indicators, production objectives, and policies.
  - Line 541: Consequently, these systematic approaches could formalise the judgmental process of stakeholder viewpoints (managers, economists, and policymakers).
  - Line 587: Therefore, researchers are encouraged to incorporate expert or value judgement to achieve their objectives. Different motivations are also observed for managers, economists, government policy makers, and academicians. Despite these individuals being committed to improving productivity, different judgements involving variable selections are presented.
- However, we believed that the paper should interest broad reach of audience both in and outside of DEA discipline. This includes student and those who a new to DEA. Therefore, this added section was written with view to be read and understand by audience regardless of their DEA knowledge background.
- We reviewed back the paper and improved in term of language, structure and related term on DEA. Proofreading was done accordingly to make the paper better. We include the certificate of Proofreading in this revised manuscript. We hope the revised paper addressed your concern

**CONCLUDING REMARKS**: We appreciate you giving us the chance to enhance our work with your insightful comments and inquiries. We made a lot of effort to take your criticism into account, and we hope that these adjustments would convince you to approve our manuscript.

Sincerely,

[A Azimatun Noor]

Corresponding Author:

A Azimatun Noor

Associate Prof Dr

Universiti Kebangsaan Malaysia

[Faculty of Medicine UKM

Level 2, Kompleks Pendidikan Perubatan Canselor Tuanku Jaafar

Jalan Yaacob Latif, Bandar Tun Razak

56000 Cheras, Kuala Lumpur

MALAYSIA]

[azimatunnoor@ppukm.ukm.edu.my]

[Tel: +603 – 9145 8693]

[Fax: +603 – 9145 8401]

Additional Contact:

M Zulfakhar Zubir

Dr

Institution/Affiliation Name

[Medical Development Division, Ministry of Health Malaysia, Putrajaya, MALAYSIA]

[dr.zulfakhar@moh.gov.my]

[Tel: +6019 – 6686415]
